# In Silico Screening and Anticancer-Apoptotic Evaluation of Newly Synthesized Thienopyrimidine/Sulfonamide Hybrids

**DOI:** 10.3390/ijms241310827

**Published:** 2023-06-29

**Authors:** Elshaymaa I. Elmongy, Faizah A. Binjubair, Ohoud Y. Alshehri, Kholoud A. Baeshen, Zaha A. Almukhalfi, Hanan A. Henidi

**Affiliations:** 1 Department of Pharmaceutical Chemistry, Faculty of Pharmacy, Helwan University, Ain Helwan, Cairo P.O. Box 11795, Egypt; 2 Department of Pharmaceutical Chemistry, College of Pharmacy, King Saud University, Riyadh 11451, Saudi Arabia; fbinjubair@ksu.edu.sa; 3 Department of Biochemistry, College of Medicine, Imam Mohammad Ibn Saud Islamic University (IMSIU), Riyadh 11564, Saudi Arabia; oyaalshehri@imamu.edu.sa; 4 Research Department, Health Sciences Research Center, Princess Nourah Bint Abdulrahman University, Riyadh 84428, Saudi Arabia; kbbaeshen@pnu.edu.sa (K.A.B.); zaalmukhalfi@pnu.edu.sa (Z.A.A.)

**Keywords:** thienopyrimidines, sulfonamides, anticancer, apoptosis

## Abstract

This work describes the design and synthesis of new hybrids of thienopyrimidine and sulfonamides. The binding affinity of the prepared compounds to FGFR-1 enzyme and caspase-3 was investigated via molecular docking. The cytotoxic effect was estimated for the synthesized compounds against human breast cancer cell lines (MCF-7 and MDA-MB231) using Doxorubicin as a reference. All the tested compounds exhibited moderate to excellent anticancer efficacy against both tested cell lines, among which **3b** and **4bi** were the best. All the synthesized compounds exhibited distinguishing selectivity index values greater than Doxorubicin. The influence of the new hybrids under inquiry was further examined on both FGFR-1 and Caspase-3. The results revealed that compound **3b** showed observed concordance between anti-proliferative activity and Caspase-3 activity. In respect to the compounds’ effect on the apoptosis, compound **3b** significantly increased the population of late apoptotic cells and necrotic cells. In silico pharmacokinetic investigation revealed that compound **3b** showed the best intestinal absorption, BBB permeability, and, along with **4bi** and **4bii**, the best CNS penetrability.

## 1. Introduction

The fibroblast growth factor (FGF) family and its four receptor tyrosine kinases (FGFR-1-4) are necessary for a variety of physiological processes, such as embryogenesis, tissue repair, tissue homeostasis, wound healing, and inflammation [1,2,3]. Fibroblast growth factor receptor 1 (FGFR-1) is involved in the regulation of cell proliferation and differentiation. FGFR-1 is also involved in other critical physiological processes and in the process of oncogenic angiogenesis; accordingly, it participates in solid tumor development [1,2,3]. FGFR-1 is considered a promising therapeutic target for the development of tumor growth inhibitors as many diseases, including cancer (such as breast, prostate, and ovarian cancers), are linked to its malfunction. Approximately 15% of breast cancers that are hormone receptor-positive and 5% of the more dangerous triple-negative breast cancers have been shown to have FGFR-1 overexpression, which is reported as frequent in breast cancer [1,2,3,4,5].

Several classes of heterocyclic rings were reported as FGFR-1 inhibitors, such as N-phenylnaphthostyril-1-sulfonamides [1], quinazolines [6], oxindoles [7], and N-phenylthieno[2,3-d]pyrimidine derivatives [5]. The latter-mentioned ring “thienopyrimidine” is reported as structurally unique, with its wide range of biological activities; it has been widely used as a scaffold in the design of compounds in the chemical sector with a variety of biologically active compounds such as enzyme inhibitors including kinases [5,8], poly-ADP ribose polymerase (PARP) inhibitors [9], and anticancer agents against some tumor cell lines [10,11,12]. In addition, its antioxidant activity [13,14,15] has been shown to be useful not only in the prevention but also in the treatment of complex diseases such as Alzheimer’s, cancer, and stroke. Additionally, in an area of anticancer drug discovery, thiourea and sulfonamide derivatives were reported to exhibit their anticancer actions via inhibiting diverse molecular targets [16], which make them promising scaffolds for novel anticancer compounds.

The foregoing facts piqued our interest, so we set out to design and synthesize thiophene/thienopyrimidine derivatives with the goal of obtaining new cytotoxic compounds, keeping in mind that the sulfonamide moiety incorporated in the designed compounds will mostly target the fibroblast growth factor receptor-1 enzyme.

**Figure 1:**
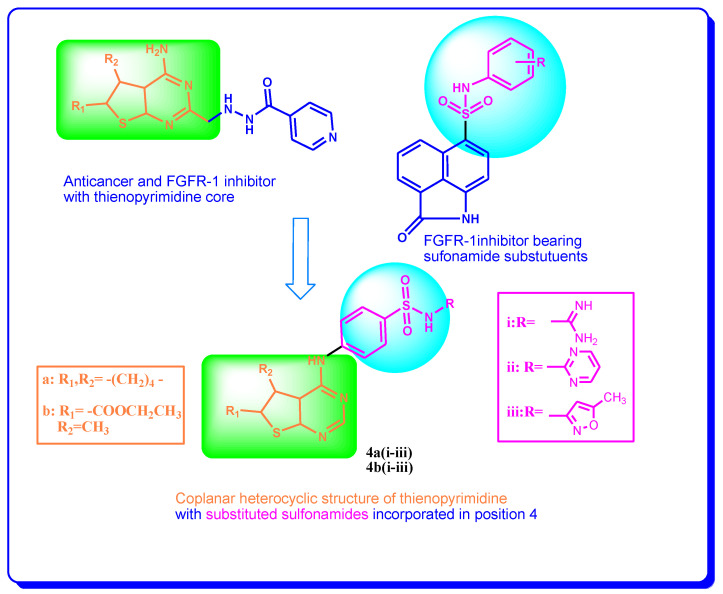
Design strategy for the synthesis of thienopyrimidine–sulfonamides hybrids [18,19].

Design strategy: The design technique involved introducing cyclohexylthieno[2,3-d]pyrimidine as a core structure (Figure 1). This was performed based on reported research that related the increased anticancer activity of thieno[2,3-d]pyrimidine with the incorporation of a lipophilic cycloalkyl ring [12,16,17]. Replacing the cyclohexyl side chain with an open ester one (R1) and a methyl group (R2) was carried out to further explore the effect of this replacement on biological activity and compare results between a cyclic side chain and the open one. Furthermore, sulfonamides had been previously incorporated in compounds resulting in promising anticancer activity, especially kinase inhibition profiles [18,19,20]. Accordingly, it prompted our interest to link the coplanar cyclic structure of thienopyrimidine with different substituted sulfonamide groups which were incorporated in the thienopyrimidine core at position 4 to afford two novel series of thienopyrimidine–sulfonamide hybrids “series **a** from **1a**–**4a**(**i**–**iii**)” and “series **b** from **1b**–**4b**(**i**–**iii**)”, aiming to explore their biological anticancer and apoptotic activities (Figure 1).

## 2. Results and Discussion

### 2.1. Synthesis

**Scheme 1:**
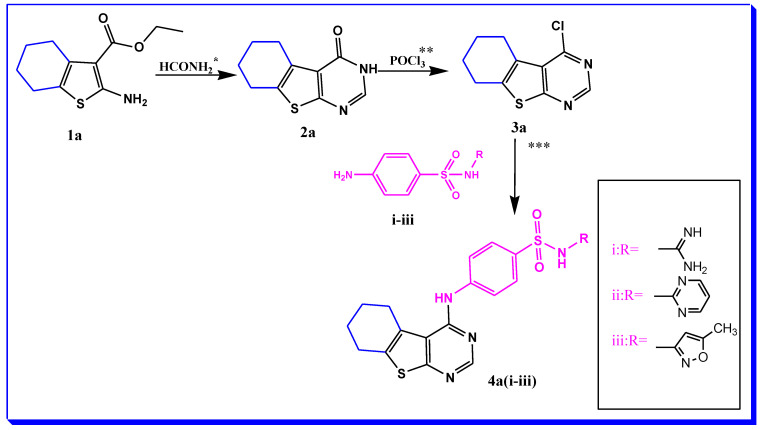
Reagents and reaction conditions: * reflux, 110 °C, 6 h; ** gentle reflux 90 °C, 8 h; *** reflux 110 °C, 12–15 h.

**Scheme 2:**
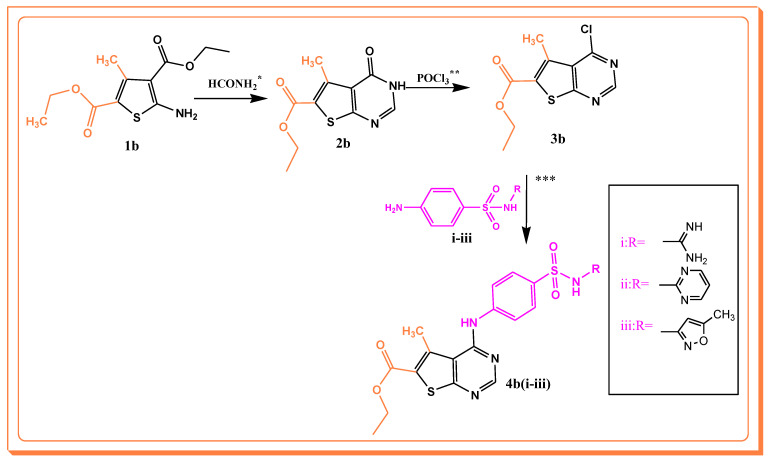
Reagents and reaction conditions: * reflux, 110 °C, 24 h; ** gentle reflux 90 °C, 10 h; *** reflux 110 °C, 12–15 h.

Synthesis pathways adopted for preparing the designed compounds **4a**(**i**–**iii**) and **4b**(**i**–**iii**) are illustrated in Scheme 1 and Scheme 2, respectively. According to the published procedures, the starting aminothiophene esters **1a** [21] and **1b** [22,23] were prepared using sulfur element, ethyl cyanoacetate, morpholine, and cycloketone or ethylacetoactate for **1a** and **1b**, respectively. Refluxing **1a**/**1b** with excess formamide as reported [21,22,23,24] gave the cycloheptathieno[2,3-d]pyrimidin-4(3H)-one derivatives **2a**/**2b**, respectively, which yielded the corresponding para chlorinated derivatives **3a**/**3b**, respectively, upon treatment with phosphorus oxychloride as described in the literature [21,25]. Refluxing the appropriate sulfonamide derivative sulfaguanidine with **3a**/**3b** in glacial acetic acid was then performed to afford the N-carbamimidoyl-4-((5,6,7,8-tetrahydrobenzo[4,5]thieno[2,3-d]pyrimidin-4-yl)amino)benzenesulfonamide/ethyl-4-((4-(N-carbamimidoylsulfamoyl) phenyl) amino)-5-methylthieno[2,3-d]pyrimidine-6-carboxylate **4a**i/**4bi**, respectively. In an attempt to explore the effect of rigidification on the activity, compounds **3a**/**3b** were refluxed with the rigid analogue of sulfaguanidine (sulfadiazine) to afford N-(pyrimidin-2-yl)-4-((5,6,7,8-tetrahydrobenzo[4,5]thieno[2,3-d]pyrimidin-4-yl)amino)benzenesulfonamide/ethyl-5-methyl-4-((4-(N-(pyrimidin-2-yl)sulfamoyl)phenyl)amino)thieno[2,3-d]pyrimidine-6-carboxylate **4aii**/**4bii**, respectively. Additionally, refluxing the p-chlorothienopyrimidine derivatives **3a**/**3b** with sulfamethoxazole was demonstrated in glacial acetic acid yielding N-(5-methylisoxazol-3-yl)-4-((5,6,7,8-tetrahydrobenzo[4,5]thieno[2,3-d]pyrimidin-4-yl)amino)benzenesulfonamide/ethyl-5-methyl-4-((4-(N-(5-methylisoxazol-3yl)sulfamoyl)phenyl)amino)thieno[2,3-d]pyrimidine-6-carboxylate **4aiii**/**4biii**, respectively (Scheme 1 and Scheme 2).

### 2.2. Biological Evaluation

#### 2.2.1. In Vitro Anti-Proliferative Activity

The cytotoxic effect of the synthesized compounds was estimated against human breast cancer cell lines (MCF-7 and MDA-MB231). The IC_50_ values of all compounds compared to Doxorubicin are summarized in Table 1. The results of cytotoxic evaluation revealed that the synthesized compounds exhibited moderate to excellent anticancer efficacy against both tested cell lines. In the estrogen receptor-positive (MCF-7) cells, compounds **3b**, **4bi**, and **4biii** demonstrated the best cytotoxic effect among all the investigated compounds, with IC_50_ 9.74 ± 0.13, 6.17 ± 1.3, and 9.54 ± 3.1 µM, respectively. The comparison of IC_50_ values against the triple-negative MDA-MB-231 cells revealed that **3b** and **4bi** had the highest potency, with IC_50_ 4.45 ± 0.31 and 8.68 ± 4.3 µM, respectively.

However, structural differences result in a distinguishing variation in cytotoxicity. The presence of three derivatives, **3b**, **4bi**, and **4biii**, possessing IC_50_ values less than 10 μM is an indication of how structure belonging to series “b” can prominently inhibit the proliferation of breast cancer cells and angiogenesis. The absence of a cyclohexyl ring and its replacement with carboxylate (open side chain) could be a contributing factor to the anticancer activity of compounds from series **b** (Table 1).

#### 2.2.2. Selectivity Assessment

Selectivity for cancer cells was investigated among the synthesized compounds to identify the compounds that have high potency towards cancer cells and low cellular toxicity in normal cells. The selectivity index (SI) was calculated using the ratio of the IC_50_ value of the selected compounds on non-cancerous cells (MCF10A) to the IC_50_ value of the compounds on both cancer cells (MCF-7 and MDA-MB-231). It has been proposed that compounds with an SI > 3 were identified to have potential selectivity towards cancer cells [26,27]. Overall, the synthesized compounds demonstrated distinguished selectivity with SI values greater than or equal to the control. Remarkably, compounds **3b** and **4bi** demonstrated the highest potential selectivity with SI values of 27.20 and 44.86 in MCF-7 cells and 59.45 and 31.90 in MDA-MB231 cells, followed by **4biii**, which demonstrated SI values of 25.62 and 13.86 in regard to MCF-7and MDA-MB231, respectively. However, compound **4aiii** displayed an SI value equivalent to the control (Table 2). These results demonstrate the potential of the synthesized compounds to selectively target cancer cells while minimizing damage to normal cells, which is a desirable characteristic in chemotherapeutic agents (Table 2).

### 2.3. Target Prediction

**Figure 2:**
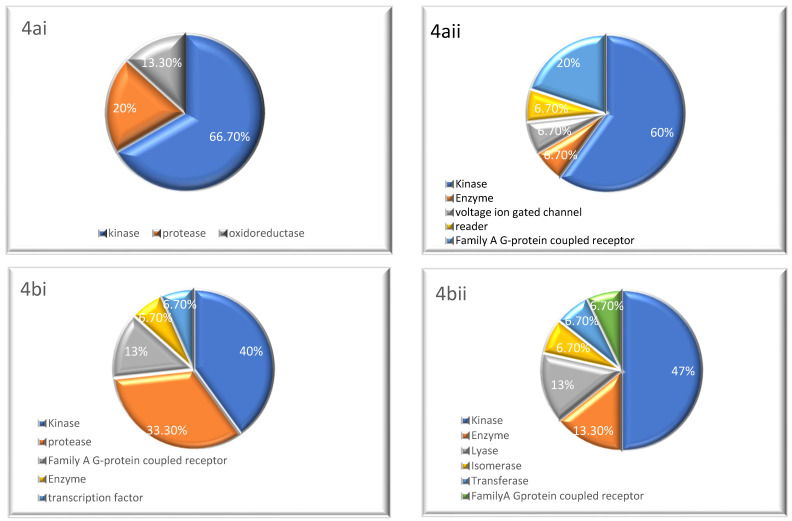
Target prediction showing compounds targeting kinase with the highest percentage.

Target prediction was performed in silico on the synthesized compounds in an attempt for exploration of the anticancer target. Compounds recorded affinity towards different targets, among them, kinases, proteases, and oxidoreductases, with percentages ranging from >60% to 6%, whereas kinase affinity was expressed in a promising value (>60% to 40%) (Figure 2).

### 2.4. Modeling

The binding affinity of the prepared compounds to FGFR-1 enzyme and caspase-3 was investigated via molecular docking. The choice of these targets was based upon a target prediction step that showed high probability for FGFR-1, while caspase-3 selection was based on our interest to explore the apoptotic activity of the newly prepared hybrids. FGFR-1 and caspase-3 proteins bounded to their ligand were downloaded from a protein data bank for modeling study with PDB codes for their crystal structures (pdb:5O49 and 7JL7, respectively). Interestingly, it was noticed that among the investigated compounds, those bearing a carboxylate side chain showed better results than those with the cyclohexyl group in both the modeling study and biological evaluations.

**Figure 3:**
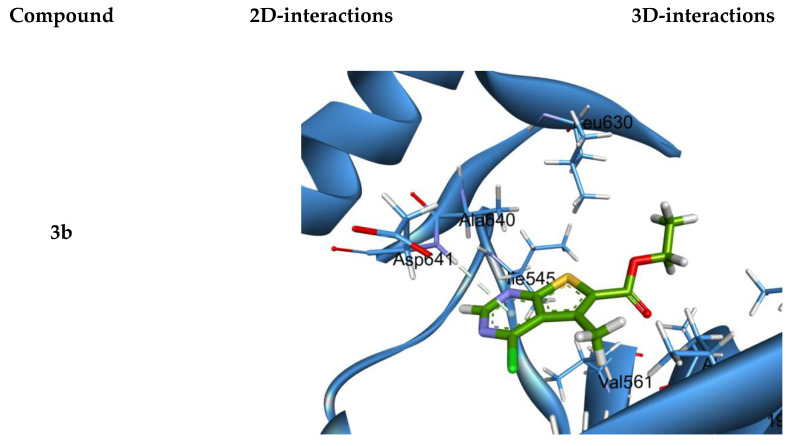

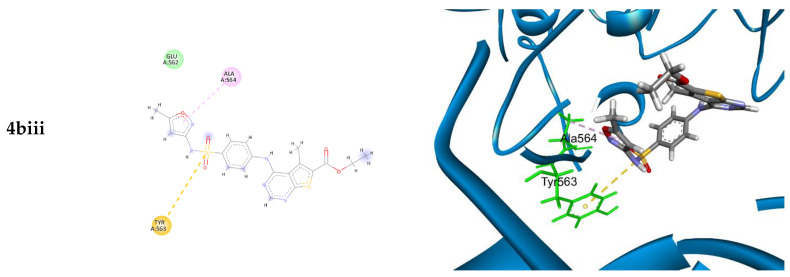
Synthesized chloro thienopyrmidine derivative **3b** which has the best biological results in green sticks and thienopyrimidine-sulfamethoxazole hybrid derivative **4biii** with the best binding energy docking score in grey sticks interacting with FGFR-1 protein crystal structure 5O49.pdb.

Regarding the FGFR-1 target protein (pdb:5O49): All compounds showed promising binding affinity to the target protein, ranging from −7.123 to −5.239. Amino acids that are commonly involved in interaction were ASP 641, LEU 484, LYS 514, and ALA 564. Compounds **3b**, **4aii**, **4bi**, and **4bii** showed interactions with the target phosphate binding pocket at ASP 641, whereas compounds **4aiii** and **4biii** revealed binding to the hinge region at ALA 564. Compounds recording the best binding affinities are illustrated in Figure 3. It worth mentioning that compound **3b** recorded the best anticancer activity, reflected in it IC_50_ values of 9.74 ± 0.13 and 4.45 ± 0.31 µM against cancer cell lines MCF-7 and MDA-MB-231, respectively, as well as its remarkable selectivity index values (SI) of 27.20 and 44.80 For MCF-7 and MDA-MB-231 cell lines, respectively. It totally fits the protein pocket (Figure 3), with hydrophobic interaction with ASP 641 at the protein phosphate binding pocket and a binding affinity of −5.239 at a perfect fit with an RMSD value of 1.0171. The binding energy and root mean square deviation (RMSD) as well as residues of amino acids incorporated in the interactions between the prepared ligands and the active site either hydrogen bonding or hydrophobic interactions are tabulated below in Table 3.

**Figure 4:**
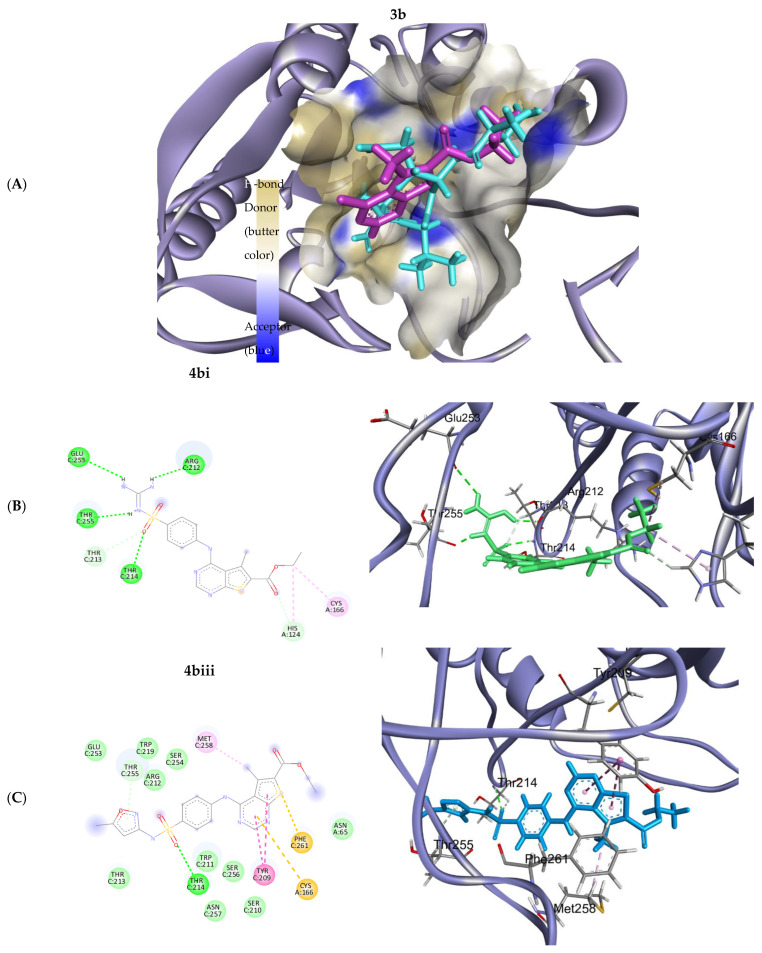
(**A**–**C**) Molecular docking interactions of compounds with best apoptotic activity in caspase enzyme assay. (**A**) Surface site view for the prepared **3b** in magenta complexed with the co-crystalized ligand of the target protein 7JL7.pdb in cyan. (**B**) Compound **4bi** in green, featuring 2D and 3D interactions with the receptor protein PDB: 7JL7. (**C**) Compound **4biii** in cyan, featuring 2D and 3D interactions with the receptor protein PDB: 7JL7.

Regarding the caspase-3 target protein (pdb: 7JL7): Binding affinity to the downloaded protein range from −6.476 to −4.059, and RMSD values range between 1.372 to 2.455A. Common amino acid residues involved in interactions are ARG 212, TRP 219, ARG 67, THR 255, and CYS 166; interestingly, these are the same residues involved in the interactions between the protein and the co-crystalized ligand, as shown in Table 4 and the overlay-complex 3D model in Figure 4. Binding energy and root mean square deviation (RMSD), as well as residues of amino acids incorporated in the interactions between the prepared ligands and the active site in addition to the types of bonding (H-bond, H–π or π–π), are tabulated below, Table 4.

### 2.5. Enzyme Specific Assay

#### 2.5.1. FGFR-1 Inhibitory Activity

After analyzing the target prediction results, it was identified that FGFR-1 had the highest probability among kinases; thus, FGFR-1 enzyme assays were conducted. Considering potency, selectivity, and affinity percentages, the inhibitory activity of all synthesized compounds against FGFR-1 was evaluated, and the results are summarized in Table 5. There was a wide range of inhibitory potencies, ranging from 131.95 ± 4.3 μM to 325.65 ± 7.4 μM. Compounds **4ai** and **4aii** had the highest FGFR-1-inhibiting activity among those tested, with IC_50_ values of 131.95 ± 4.3 and 119.43 ± 5.6 μM, respectively. However, it was challenging to correlate the anticancer effect of the drugs that were examined to the inhibition of FGFR-1 alone owing to the compounds’ modest inhibitory action against FGFR-1. This may imply the presence of alternate underlying mechanisms other than interacting with FGFR-1 to boost the anticancer effects of the compounds that were tested, or it might suggest that another kinase enzyme might be involved in the mechanism of action.

#### 2.5.2. Caspase-3 Activity

**Figure 5:**
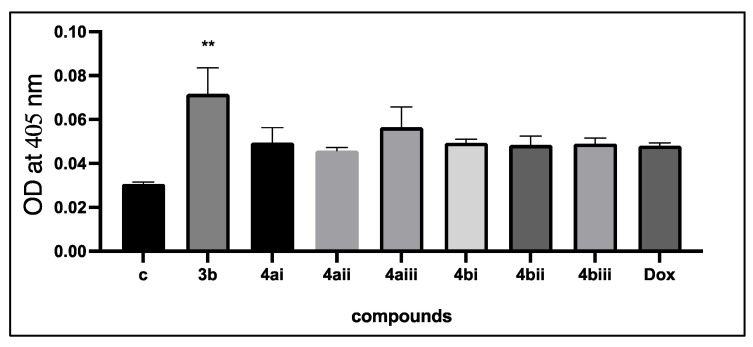
Caspase-3 activity was assessed using the Caspase-3 Colorimetric Assay kit. The data are presented as the mean ± SD of three independent experiments. ** *p* < 0.01 vs. the untreated group.

To further explore the cytotoxicity of the prepared hybrids, their influence on Caspase-3 activity was examined. MDA-MB-231 cells were incubated with the predetermined IC_50_s of tested compounds for a duration of 24 h. The results revealed that all compounds induced Caspase-3 activity in comparison to control cells, with fold change ranging from 1.4 to 2.3 folds (Figure 5). However, compound **3b** showed 2.3-fold concordance between anti-proliferative activity and the Caspase-3 activity, (Figure 5, *p* < 0.01).

### 2.6. Annexin V/Propidium Iodide (PI) Flow Cytometric Analysis

**Figure 6:**
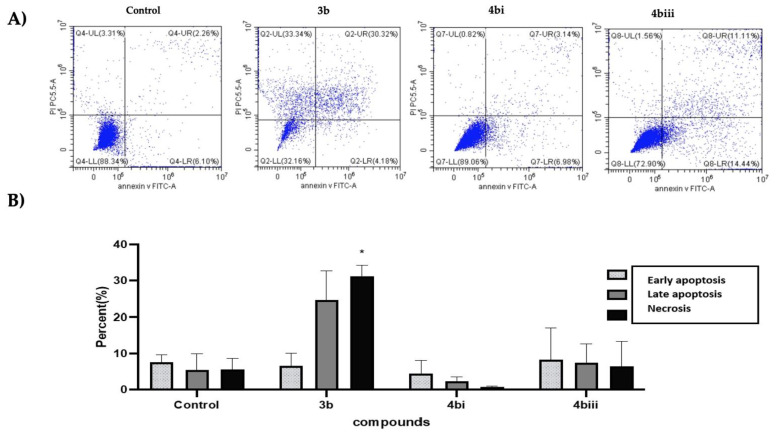
(**A**) representative flow cytometry results for apoptosis detected at 48 h after treatment with **3b**, **4bi**, and **4biii**. (**B**) Percentage of Annexin V-FITC/PI-positive apoptotic cells. Data represent the mean ± SD from three independent experiments. * *p* < 0.01.

Apoptosis and necrosis are two routes that make up the overall division of the cell death pathway carried out by anticancer agents. A double labeling flow cytometry assay using Annexin V-FITC/propidium iodide was performed to identify which mechanism, necrosis, or apoptosis is responsible for cell death [28]. In accordance with the IC_50_ values obtained from Table 1, compounds **3b**, **4bi**, and **4biii** were selected to be examined further in respect to their effect on the apoptosis (Figure 6). Among all compounds examined, compound **3b** significantly increased the population of late apoptotic cells and necrotic cells 4.8 and 5.5 fold, respectively. In addition to demonstrating that the calculated IC_50_ value is indicative of cytotoxic effects with/without moderate antiproliferative activity, this explains the cell killing effects attributed to compound **3b** against MDA-MB231 cells.

### 2.7. Insilico Investigation

#### 2.7.1. Investigation of Drug Likeness and Physicochemical Properties

In silico screening of pharmacokinetic and physicochemical properties was performed on the biologically evaluated thienopyrimidine-sulfonamide hybrids **3b**, **4a**(**i**–**iii**), and **4b**(**i**–**iii**). All the tested compounds recorded log *p* < 5, which indicated their high cell membrane tolerability [29]. In addition, the topological polar surface area (TPSA) values range from 146.38 to 196.77. The number of hydrogen bond acceptors (HBAs) < 10 acceptors, while the number of hydrogen bond donors (HBDs) are either two or four, and their molecular weights (MW) are less than 500, which is consistent with Lipinski’s rule of five (Table 6).

**Figure 7:**
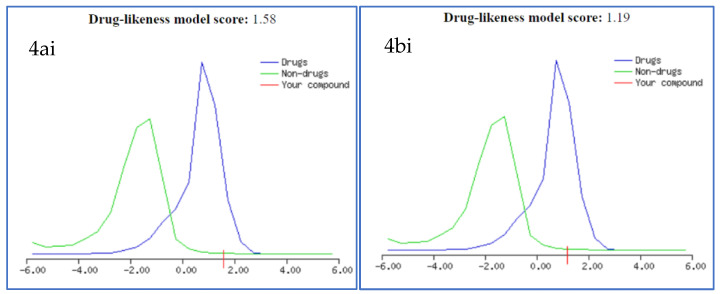
Illustration of the best drug likeness scores: thienopyrimidine with sulfaguaidine-incorporated hybrids (**4ai** and **4bi**).

According to reported literature [30,31], compounds with a positive drug likeness score are considered good drug candidates. As shown in Table 6, all the investigated compounds except **3b** expressed positive drug likeness values ranging from 0.73 to 1.58, among which the N-carbamimidoylcyclohexathieno[2,3-d]pyrimidine benzenesulfonamide derivative **4ai** and Ethyl-4-((4-(N-carbamimidoylsulfamoyl)phenyl)amino)-5-methylthieno[2,3-d]pyrimidine-6-carboxylate **4bi** recorded the best scores (1.58 and 1.19, respectively; Figure 7). As shown in Table 6, all the prepared compounds expressed no Lipinski’s rule violations [32] and are considered promising “drug-like” molecules.

#### 2.7.2. Pharmacokinetics In Silico Assessment for the Prepared Hybrids

ADMET analysis is especially helpful in simplifying clinical trials, especially in the early stage of drug design. Intestinal absorption, skin sensitization, and oral bioavailability are the absorption parameters considered in drug discovery [33]. An intestinal absorption score >30% indicates perfect absorbance. As tabulated in Table 7, all compounds recorded intestinal absorption of significantly more than 30%, with a minimum of 64% for compound **4bi** and a maximum value of 96% demonstrated by **3b**, which reflects an excellent absorbance rate. A compound is known to have a relatively low skin permeability if it has log Kp > −2.5 [33]. The prepared compounds revealed good skin permeability, with a skin permeability average score around −2.88 cm/h. Compounds are considered to possess high human colon adenocarcinoma (Caco2) permeability when they record Caco2 values > 0.9 [33]; however, the currently investigated thienopyrimidine sulfonamides hybrids revealed low human colon adenocarcinoma permeability as all compounds showed values < 0.9, except **3b**, which showed Caco2 permeability of 1.324.

To investigate the compounds’ distribution in silico, volume of distribution (VDss), blood–brain barrier (BBB) membrane permeability, and CNS permeability were assessed. With VDss values, a larger distribution volume was observed in compound **4ai**, with 0.397 log L/kg (Table 7). Log BB value for compounds will reflect low BBB if <−1 as reported [33]. Among the tested compounds, **3b** expressed the best and most promising permeability of the BBB membrane with a score of 0.184. Log PS values for CNS permeability range from −2.18 to −3.24, and since low CNS permeability is reported if log PS is <−3 [33], compounds **3b**, **4bi**, and **4bii** are considered to have promising CNS permeability, while compounds **4a**(**i**–**iii**) and 4biii recorded log PS < −3, indicating impenetrability.

Hepatic and renal clearance were used to examine overall drug clearance. Total clearance calculates the drug’s concentration in the body utilizing the elimination rate. Compounds’ excretion rate is demonstrated in log(mL/min/kg) in Table 7. The anticipated scores for ADMET analysis are summarized in Table 7.

## 3. Materials and Methods

### 3.1. Chemistry

All NMR analyses were performed with the Bruker magnet system 400′54 Ascend/R (USA) using 400 MHz and 100 MHz for ^1^H-NMR and ^13^C-NMR. Mass spectrum was performed on Direct Inlet part to mass analyzer in GCMS model with ISQ single quadrupole thermoscientific Electron Impact mode (UK). Melting points (m.p.) were determined using the Stuart scientific melting point apparatus and are uncorrected.

Compounds **1** and **2** were prepared according to the respectively reported procedures in [34] (Scheme 1). Compounds **1a** and **1b** were prepared following Gewald reaction between sulfur powder, morpholine, ethylcyanoacetate, and either cyclohexanone or ethylacetoacetate to yield **1a** and **1b**, respectively. Compounds **2a** and **2b** were obtained upon reacting the aminocarboxylate esters **1a** and **1b**, respectively, with formamide, which is then chlorinated to the chloride derivatives **3a** and **3b** using phosphorus oxychloride [21].

General procedures for synthesis of **4a**(**i**–**iii**) and **4b**(**i**–**iii**): Equimolar amounts of the chloride derivatives **3a**/**3b** and the appropriate sulfonamide, namely, sulfaguanidine, sulfadiazine, and sulfamethoxazole, were refluxed in glacial acetic acid (15 mL) for 15 h; the reaction mixture was then left to cool at R.T and then poured onto ice water. The formed solid was filtered and crystalized from absolute ethanol to yield **4a**(**i**–**iii**) and **4b**(**i**–**iii**) series, respectively.

**4ai: N-carbamimidoyl-4-((5,6,7,8-tetrahydrobenzo[4,5]thieno[2,3-d]pyrimidin-4-yl)amino) benzenesulfonamide**

m.p > 250 °C and yield 75%. EI–MS *m*/*z* for: C_17_H_18_N_6_O_2_S_2_ (402). ^1^H NMR (400 MHz, DMSO-d6) ppm: δ 1.24–2.98 (m, 8H, cyclohexane), 4.9 (s, 1H, D_2_O exchangeable NH), 5.50 (s, 2H, exchangeable, NH_2_), 6.90–7.12 (m, aromatic-4H), 8.60 (s.1H, C2-pyrimidine), 12.00 (s, 1H, D_2_O exchangeable NH). ^13^C-NMR (DMSO, d6) ppm δ: 20.2, 21.5, 23.0, 23.2, 25.8, 119.7, 124.3, 125.1, 126.5, 126.5, 129.2, 129.8, 139.2, 141.4, 155.6, 156.1, 164.8.

**4aii:N-(pyrimidin-2-yl)-4-((5,6,7,8-tetrahydrobenzo[4,5]thieno[2,3-d]pyrimidin-4-yl)amino)benzenesulfonamide**

m.p. > 250 °C, yield 70%. EI–MS *m*/*z* for: C_20_H_18_N_6_O_2_S_2_ (438). ^1^H NMR (400 MHz, DMSO-d6) ppm: δ 1.24–2.98 (m, 8H, cyclohexane), 5.50 (s, 1H, exchangeable, NH), 6.34 (s, H, C5-pyrimidine of sulfadiazine), 7.00–7.12 (m, aromatic-4H), 8.39 (m, 3H, C2-of thienopyrimidine and C4,C6-pyrimidine of sulfadiazine), 12.00 (s, 1H, D_2_O exchangeable NH). ^13^C-NMR (DMSO, d6) ppm δ: 20.2, 21.5, 23.0, 23.2, 25.8, 110.3, 113.7, 119.7, 124.3, 127.5, 128.2, 128.8, 129.2, 137.4, 145.6, 146.1, 153.2, 156.3, 157.9, 169.8.

**4aiii:N-(5-methylisoxazol-3-yl)-4-((5,6,7,8-tetrahydrobenzo[4,5]thieno[2,3-d]pyrimidin-4-yl)amino)benzenesulfonamide**

m.p. > 250 °C, yield 72%. EI–MS *m*/*z* for: C_20_H_19_N_5_O_3_S_2_ (441). ^1^H NMR (400 MHz, DMSO-d6) ppm: δ 1.20–3.00 (m, 3H, CH_3_ at C3-isoxazole and 8H, cyclohexane), 4.11 (s, 1H, isoxazole), 5.50 (s, 1H, exchangeable NH), 6.90–7.12 (m, aromatic-4H), 12.00 (s, 1H, D_2_O exchangeable NH).^13^C-NMR (DMSO, d6) ppm δ: 19.3, 20.4, 22.6, 23.5, 25.4, 95.1, 114.7, 116.8, 117.8, 126.9, 127.5, 128.0, 129.1, 139.2, 145.1, 146.4, 152.4, 153, 159.2, 162.8.

**4bi:Ethyl-4-((4-(N-carbamimidoylsulfamoyl)phenyl)amino)-5-methylthieno[2,3-d]pyrimidine-6-carboxylate**

m.p. 154–156 °C, yield 72%. EI–MS *m*/*z* for C_17_H_18_N_6_O_4_S_2_ (434). ^1^H NMR(400 MHz, DMSO-d6) ppm: δ 1.28 (t, 3H, CH_3_), 2.44 (s,3H, CH_3_), 4.16 (q, 2H, CH_2_), 4.9 (s, 1H, D_2_O exchangeable NH), 5.50 (s, 2H, exchangeable, NH_2_), 7.96 (m, 4H aromatic), 8.74 (s, 1H, CH), 11.0 (s, 1H, D_2_O exchangeable NH). ^13^C NMR(DMSO-d6) ppm: δ 10.4, 14.2, 61.2, 116.6, 118.9, 119.6, 128.0, 128.4, 129.6, 144.6, 146.0, 148.6, 152.9, 153.7, 156.8, 158.9, 163.3.

**4bii:Ethyl-5-methyl-4-((4-(N-(pyrimidin-2-yl)sulfamoyl)phenyl)amino)thieno[2,3-d]pyrimidine-6-carboxylate**

m.p. 238–240 °C, yield 78%. EI–MS *m*/*z* C_20_H_18_N_6_O_4_S_2_ (470). ^1^H NMR(400 MHz, DMSO-d6) ppm: δ 1.28 (t, 3H, CH_3_), 2.44 (s,3H, CH_3_), 4.16 (q, 2H, CH_2_), 4.9 (s, 1H, D_2_O exchangeable NH), 7.96 (m, 4H aromatic), 5.50 (s, 2H, exchangeable, NH_2_), 7.96 (m, 4H aromatic), 8.39–8.51 (m, 2H, C4,C6-diazine), 8.74 (s, 1H, CH), 11.00 (s, 1H, D_2_O exchangeable NH). ^13^C NMR: δ 10.4, 14.2, 20.1, 61.2, 117.4, 119.4, 119.6, 128.0, 128.2, 128.6, 141.6, 146.0, 146.6, 150.3, 153.8, 154.9, 156.8, 158.3, 160.9, 163.3.

**4biii:Ethyl-5-methyl-4-((4-(N-(5-methylisoxazol-3-yl)sulfamoyl)phenyl)amino) thieno[2,3-d]pyrimidine-6-carboxylate**

m.p. 138–140 °C, yield 64%. EI–MS *m*/*z* forC_20_H_19_N_5_O_5_S_2_ (473). ^1^H NMR (400 MHz, DMSO-d6) ppm: δ 1.28 (t, 3H, CH_3_), 2.44 (m, 3H, CH_3_ and 3H CH3 at C3-isoxazole), 3.50 (s, 1H isoxazole) 4.0 (s, 1H, D_2_O exchangeable NH), 4.16 (q, 2H, CH_2_), 7.96 (m, 4H aromatic), 7.96 (m, 4H aromatic), 8.49 (s, 1H, CH), 11.00 (s, 1H, D_2_O exchangeable NH). ^13^C NMR: δ 10.4, 12.9, 14.2, 61.2, 100.1, 115.4, 116.4, 116.6, 128.2, 128.2, 129.6, 144.3, 146.3, 148.7, 150.3, 153.8, 154.9, 158.3, 160.9, 163.3.

### 3.2. Biological Evaluation

#### 3.2.1. Anti-Proliferative Activities towards Breast Cancer Cells

MTT (3-[4,5-dimethylthiazol-2-yl]-2,5 diphenyltetrazolium bromide) assay was used to study the anticancer activity of all derivatives against breast cancer cells MCF-7and MDAMB231 (obtained from ATCC, Manassas, VA, USA). Doxorubicin (Sigma, St. Louis, MO, USA) was taken as a positive standard in this study. Briefly, cells plated onto 96-well plates (5000 cells/well) were allowed to adhere and form a semi-confluent monolayer. After 24 h, the cells were treated with different concentrations of derivatives (0–100 µM) for 72 h. After the exposure, cytotoxicity was assessed following the MTT Assay Kit protocol (Abcam, Cambridge, UK) as reported earlier [35].

#### 3.2.2. Selectivity Index (SI)

To determine if the new derivatives have selectivity towards cancer cells, the cytotoxic effect on non-tumorigenic cell line MCF10A was evaluated. The selectivity ratio in non-cancerous cells (MCF10A) versus cancer cells (MCF-7 and MDAmb231) was calculated as reported in [26,27] using the following equation: SI = IC_50_ of normal cells/IC_50_ of cancer cells.

#### 3.2.3. FGFR-1 Inhibitory Activity

To determine basal FGFR-1 activity, a FGFR-1 ELISA Kit was used according to the protocol of the manufacturer (G-Biosciences, Saint Louis, MO, USA). In brief, MDA-MB-231 cell lysates (100 ng/mL) were diluted and added to the Eliza plate. After 90 min of incubation at 37 °C, 100 μL Biotinylated Detection Antibody was added and incubated for 60 min. After washing twice each with washing buffer, 100 μL ELISA Detection Reagent was added to each well and incubated for 30 min. After washing, 90 μL of Detection Substrate (TMB) was added to visualize the enzymatic reaction. TMB was catalyzed to produce a blue color that changed into yellow after adding 50 μL of acidic stop solution. The density of yellow is directly proportional to the concentration of FGFR-1 captured on the plate using a Varioskan TM LUX multimode microplate reader (Thermo Scientific, Waltham, MA, USA) [36].

#### 3.2.4. Caspase-3 Activity Assay

Caspase-3 activity was assessed with a colorimetric assay based on the hydrolysis of acetyl–Asp–Glu–Val–Asp p-nitroanilide (Ac-DEVD-pNA) by Caspase-3 and releasing p-nitroaniline (pNA). MDA-MB-231 cell lysates (100 ng/mL) were diluted and subjected to analysis via Caspase-3 Colorimetric Assay Kit (Sigma-Aldrich, Saint Louis, MO, USA) based on the protocol of the manufacturer. The reaction for color development was held at 37 °C for 2 h, and the value of OD405 was determined using a Varioskan TM LUX multimode microplate reader (Thermo Scientific, Waltham, MA, USA). Each caspase-3 activity was expressed as a value of OD405.

#### 3.2.5. Evaluation of Apoptosis by Annexine V

Apoptosis was detected after 48 h of cell culture using V-FITC apoptosis detection kit (BD Pharmingen TM, Franklin Lakes, NJ, USA). MDA-MB-231 cells were treated with the predetermined IC_50_s of the most potent compounds for 24 h, and the untreated group was included as a control group. After treatment, cells were collected, washed with ice-cold PBS and centrifuged at 200× *g* for 5 min. The cell pellet was suspended in 200 μL of annexin V-FITC/PI solution for 15 min in the dark. The stained and Annexine V propidium iodide negative cells were evaluated via CytoFLEX flow cytometry (Beckman Coulter, Brea, CA, USA). Ten thousand cells (gated events) were captured for each sample. The mean fluorescence intensity was analyzed with Cytoexpert (Beckman Coulter).

### 3.3. Target Prediction

Scanning for targets was performed using Swiss Target [37]; smiles were copied to the free available software accessed on 6 September 2022, where calculations were run for target prediction and indications.

### 3.4. Molecular Modeling

Modeling studies were performed with the aid of molecular operating environment MOE [38] and Discovery Studio 2021 software v21.1.0.20298. Two proteins were downloaded for modeling study from the protein data bank (pdb:5O49 and 7JL7). The crystal structure FGFR-1 bound to its co crystallized ligand was downloaded from Protein Data Bank (PDB code:5O49), as was caspase-3 with its co-ligand at chain F [39,40]. All compound structures were built on MOE builder, corrected, energy minimized, and saved as mol2 format. Induced fit was the selected protocol. Ligand was set as site of docking in caspase-3, while dummy atoms were set as a placement guide for FGFR-1 docking. The gradient for energy minimization was set as default at 0.05, and the applied force field was also applied as default MMFF94X.

### 3.5. Statistical Analysis

The data were analyzed using Prism^®^ for Windows, ver. 9.00 (GraphPad Sofware Inc., La Jolla, CA, USA) and presented as mean ± S.D. The statistical tool used for testing the significance in this study was Analysis of Variance (ANOVA) with the Least Significant Difference (LSD) post hoc test. The software used for the analysis was SPSS^®^ for Windows, version 17.

### 3.6. In Silico Drug Likeness and Pharmacokinetics Investigation

In silico assessment was performed on the selected compounds using Molsoft (www.molsoft.com accessed on 22 September 2022), preADMET (https://preadmet.bmdrc.kr/ accessed on 13 March 2023), and Swiss ADME [29].

## 4. Conclusions

This work describes the biological evaluation of new synthesized hybrids of thienopyrimidine and sulfonamides. Docking studies were applied to calculate the binding affinity scores of the synthesized hybrids to FGFR-1 enzymes and caspase-3. All compounds showed promising binding affinity to the FGFR-1 target protein, ranging from −7.123 to −5.239, whereas binding affinity to Caspase-3 ranged from −6.476 to −4.059. The cytotoxic effect of the synthesized compounds was estimated against two human breast cancer cell lines (MCF-7 and MDA-MB231) using Doxorubicin as a reference. All the tested compounds exhibited moderate to excellent anticancer efficacy against both tested cell lines, among which **3b** and **4bi** were the best. In MCF-7 cells, compound **4bi** displayed the highest cytotoxic activity among all the tested compounds, with IC_50_ of 6.17 ± 1.3 µM, while compound **3b** recorded the lowest IC_50_ value against MDA-MB-231 cells with IC50 of 4.45 ± 0.31 µM. For further exploration of the anticancer activity of the tested compounds, their selectivity was examined through calculating their selectivity index using normal MCF-10A cells. All the synthesized compounds exhibited distinguishing selectivity index values greater than the Doxorubicin. To further dissect the cytotoxic effects of the compounds under investigations, the influence of these compounds on both FGFR-1 and Caspase-3 activity was examined. The results revealed that compound **3b** showed concordance between anti-proliferative activity and Caspase-3 activity. In respect to compounds’ effect on apoptosis, compound **3b** significantly increased the population of late apoptotic cells and necrotic cells by 4.8 and 5.5 times, respectively. Structural changes produced a distinct difference in cytotoxicity. Three derivatives from the series “b” structure, **3b**, **4bi**, and **4biii**, all have IC_50_ values below 10 µM, which shows how effectively they can block angiogenesis and the proliferation of breast cancer cells. The absence of a cyclohexyl ring and its replacement with a carboxylate (open side chain) may contribute to the anticancer activity of compounds from series b. In silico pharmacokinetic assessment revealed that all compounds had no violations to Lipinski’s rule. Drug likeness investigation showed that compounds **4a**(**i**–**iii**) and **4b**(**i**–**iii**) are drug-like, in particular compound **4ai**, which has a guanidine group engaged with the thienopyrimidine core that recorded the highest positive score of 1.58.

## Figures and Tables

**Table 1:** Anti-proliferative activity of derivatives after 72 h of exposure against MCF-7 and MDA-MB-231 cancer cell lines, as well as towards breast MCF-10A normal cells.

Compound No.	IC_50_ * (µM)		
	MCF-7	MDA-MB-231	MCF-10A (Non-Tumorigenic Cells)
**3b**	9.74 ± 0.13	4.45 ± 0.31	264.9 ± 4.2
**4ai**	35.79 ± 1.2	40.17 ± 2.7	457.9 ± 12.3
**4aii**	22.29 ± 3.1	20.19 ± 1.5	>1000
**4aiii**	67.07 ± 15.7	68.345 ± 4.6	88.45 ± 10.1
**4bi**	6.17 ± 1.3	8.68 ± 4.3	277.05 ± 7.9
**4bii**	13.97 ± 8.2	15.57 ± 0.1	229.9 ± 24.1
**4biii**	9.54 ± 3.1	17.64 ± 2.3	244.5 ± 1.9
**Doxorubicin**	1.6 ± 2.2	2.2 ± 0.3	2.8 ± 1.2

* IC_50_ values are expressed as mean ± S.D. for three independent observations.

**Table 2:** The selectivity index (SI) which represents IC_50_ for non-tumorigenic immortalized cells MCF 10A/IC_50_ for cancerous cell lines after 72 h of exposure to the tested compounds.

Compound No.	MCF-7	MDA-MB231
**3b**	27.20	59.45
**4ai**	12.79	11.40
**4aii**	ND ^a^	ND ^a^
**4aiii**	1.32	1.29
**4bi**	44.86	31.90
**4bii**	16.45	14.77
**4biii**	25.62	13.86
**Doxorubicin**	1.75	1.27

^a^ ND: Not determined.

**Table 3:** Binding energy scores along with RMSD values at FGFR-1 binding site.

Compound	Protein-Pdb Code	Binding Energy	RMSD	Amino Acids Involved in Interaction	Types of Interaction
**3b**	5O49	−5.239	1.0171	ASP 641	*Pi*-H
**4ai**	5O49	−6.866	1.873	GLU 531 LEU 484 GLY 567	H-donor *Pi*-H *Pi*-H
**4aii**	5O49	−5.989	1.905	ASP 641 LYS 514 GLY 567	H-donor H-acceptor *Pi*-H
**4aiii**	5O49	−7.091	2.191	ALA 564	H-acceptor
**4bi**	5O49	−6.443	2.093	ASP 641 TYR 563 LEU 484	H-donor H-acceptor *Pi*-H
**4bii**	5O49	−6.365	1.781	ASP 641 TYR 563 LEU 484 LYS 514	H-acceptor H-acceptor *Pi*-H *Pi*-cation
**4biii**	5O49	−7.123	1.776	ALA 564 TYR 563	*Pi*-H *Pi*-sulfur

**Table 4:** Binding energy scores along with RMSD values at Caspase-3.

Compound	Protein Pdb Code	Binding Energy	RMSD	Amino Acids Involved in Interaction	Types of Interaction
**3b**	7JL7	−4.059	1.7578	ARG 212	H-donor
**4ai**	7JL7	−6.157	1.372	SER 123 TYR 209 TYR 209 ARG 67	H-donor H-*pi* H-*pi* *Pi*-cation
**4aii**	7JL7	−6.245	2.455	ARG212	H-acceptor
**4aiii**	7JL7	−5.803	2.128	THR 255	H-acceptor
**4bi**	7JL7	−5.925	2.166	THR 255 THR 214 ARG212 GLU 253 CYS 166 HIS 124	H-acceptor H-donor H-bond H-bond H-bond *Pi*-H
**4bii**	7JL7	−4.981	1.978	PRO 66 ASN 257 ASN 65	H-donor H-acceptor *Pi*-H
**4biii**	7JL7	−5.939	1.801	THR 214 TYR 209 MET 258 CYS 166 PHE 261	H-donor π-π π-π π-sulfur π-sulfur

**Table 5:** IC_50_ values in μM reflecting FGFR-1 inhibitory activity of the selected compounds.

Compound No.	IC_50_ (μM)
**3b**	179.15 ± 5.6
**4ai**	131.95 ± 4.3
**4aii**	119.43 ± 5.6
**4aiii**	159.2 ± 5.3
**4bi**	325.65 ± 7.4
**4bii**	289.166 ± 12.0
**4biii**	168.05 ± 8.3
**Doxorubicin**	53.09 ± 3.2

**Table 6:** Physicochemical and lipophilicity in silico screening for the prepared hybrids.

	MW	HBA	HBD	MR	TPSA	iLog *p*	Lipinski Violations	Drug Likeness
**3b**	256.71	4	0	63.48	80.32	2.6	0	−0.34
**4ai**	402.49	5	4	106.58	170.47	1.51	0	1.58
**4aii**	438.53	6	2	117.09	146.38	2.66	0	1.03
**4aiii**	441.53	6	2	116.53	146.63	2.59	0	1.08
**4bi**	434.49	7	4	110.21	196.77	1.75	0	1.19
**4bii**	470.52	8	2	120.71	172.68	2.78	0	0.88
**4biii**	473.53	8	2	120.15	172.93	3.1	0	0.73

**Table 7:** ADMET in silico screening results for the prepared theinopyrimidine–sulfonaimde hybrids.

**Cpd. No.**	**ABSORPTION**				
	Water solubility (log mol/L)	Caco2 permeability *	Intestinal absorption (human)%	log Kp **	
**3b**	−3.458	1.324	96.254	−2.895	
**4ai**	−3.037	0.207	77.346	−2.735	
**4aii**	−4.457	0.838	88.833	−2.892	
**4aiii**	−4.743	0.72	95.347	−2.901	
**4bi**	−3.004	−0.261	64.447	−2.735	
**4bii**	−4.108	0.329	75.934	−2.85	
**4biii**	−4.387	0.211	82.448	−2.871	
**Cpd. No.**	**DISTRIBUTION**			**EXCRETION**	**TOXICITY**
	VDss *** (human)	BBB **** permeability	CNS permeability	Total Clearance	Max. tolerated dose (human)
**3b**	−0.359	0.184	−2.91	0.337	0.84
**4ai**	0.397	−0.876	−2.655	−0.07	0.829
**4aii**	0.137	−0.906	−2.309	−0.097	−0.546
**4aiii**	0.176	−0.852	−2.182	−0.071	−0.611
**4bi**	0.372	−1.323	−3.195	−0.107	0.602
**4bii**	−0.277	−1.583	−3.249	0.031	−0.561
**4biii**	−0.27	−1.529	−2.722	0.057	−0.612

* CACO-2: permeability through cells derived from human colon (log Papp in 10^−6^ cm/s); ** log Kp: Skin Permeability; *** VDss: volume of distribution; **** BBB: blood–brain barrier penetration.

## Data Availability

Not applicable.
